# Supplementary material for: X-ray radiation damage cycle of solvated inorganic ions
Source: Nat Commun. 2024 May 30;15:4594. doi: 10.1038/s41467-024-48687-2 (PMC11139941; doi:10.1038/s41467-024-48687-2)
Supplement: Supplementary file 1 — Supplementray Information [file 41467_2024_48687_MOESM1_ESM.pdf]

Supplementary Information – X-ray radiation damage cycle of solvated inorganic ions

Dana Bloß[1]*, Florian Trinter[2,3], Isaak Unger[4], Christina Zindel[1], Carolin Honisch[1], Johannes Viehmann[1], Nils Kiefer[1], Lutz Marder[1], Catmarna Küstner-Wetekam[1], Emilia Heikura[1], Lorenz S. Cederbaum[5], Olle Björneholm[4], Uwe Hergenhahn[2], Arno Ehresmann[1], and Andreas Hans[1+]

*dana.bloss@uni-kassel.de
+hans@physik.uni-kassel.de

[1] Institute of Physics und Center for Interdisciplinary Nanostructure Science and Technology (CINSaT), University of Kassel, Heinrich-Plett-Straße 40, 34132 Kassel, Germany
[2] Fritz-Haber-Institut der Max-Planck-Gesellschaft, Faradayweg 4-6, 14195 Berlin, Germany
[3] Institut für Kernphysik, Goethe-Universität Frankfurt, Max-von-Laue-Straße 1, 60438 Frankfurt am Main, Germany
[4] Chemical and Biomolecular Physics, Department of Physics and Astronomy, Uppsala University, Box 516, 75120 Uppsala, Sweden
[5] Theoretical Chemistry, Institute of Physical Chemistry, University of Heidelberg, Im Neuenheimer Feld 229, 69120 Heidelberg, Germany

Supplementary Note 1

A major challenge for the interpretation of electron spectra measured from the liquid phase is the structureless background of low-kinetic-energy electrons[1–5]. The approach followed in the present work is the detection of the electrons in coincidence. One major advantage of coincidence techniques is given by the application of coincidence conditions: if one electron and its kinetic energy is known (e.g., an Auger electron or photoelectron), all electrons emitted in coincidence with it can be identified. Additionally, all contributions to the spectrum are removed which result from events in which that known electron underwent inelastic scattering. Detection of several electrons in coincidence was afforded by choosing a spectrometer capable of collecting a large solid angle (magnetic bottle spectrometer).

In our experiments, double- and triple-electron coincidences were analyzed. We set coincidence conditions for the first detected electron, which was either a photoelectron or an Auger electron, the kinetic energies of which are well-defined in both cases. Here, the term first refers to flight-times of the electrons in our spectrometer, which are orders of magnitude larger than the temporal delay between photoelectron, Auger electron and ICD/ETMD electrons. The spectra of the second (and for triple coincidences also third) detected electron were then further analyzed. An exemplary spectrum of the second electron filtered for a distinct first electron (here Mg^{2+} 2p photoelectron) is shown as green solid line in Fig. 3(a) of the main article, exhibiting weakly structured features as well as an increasing background towards lower kinetic energies. To enhance the visibility of distinct features the background needs to be subtracted. Two variants of background subtraction were applied: (1) from the spectrum of the second (or third) electron, obtained through the coincidence filter, we subtracted the unfiltered second (or third) detected electron from the same measurement [e.g., gray dashed curve in Fig. 3(a)]. (2) From the spectrum of the second (or third) electron, obtained through the coincidence filter, we subtracted a spectrum using the unfiltered second (or third) electron but from a reference measurement with pure water. Both approaches assume that the total number of low-energy electrons occurring due to inelastic scattering is significantly higher than that originating from the structured features, i.e., that the unfiltered spectra resemble the background of scattered electrons well. Before subtraction the spectra need to be normalized. For the normalization

two procedures were applied: normalization to curve maximum or to curve integral. Both methods showed no qualitative difference in the results, which is why only the former is used for all spectra presented here.
Due to the normalization and subtraction process no conclusions about the relative or absolute intensities of different features are possible in the difference spectra. The low-energy cutoff of the difference spectra results from the applied retardation and jet bias. We confirmed that in all presented spectra the different normalization procedures and subtraction of different background references did only alter the absolute and relative intensities of the observed features, but did not affect the overall structure of the spectra and energetic positions.

Supplementary Note 2

The investigation of the Al^{3+} 1s ionization of a 2 M $AlCl_3$ solution with a hemispherical analyzer was already presented by some of the authors and reported in Ref. [6]. As a proof-of-principle for our method and analysis procedure, we reproduced the measurement for a 2 M $AlCl_3$ solution choosing a similar exciting-photon energy around 1572 eV with the magnetic bottle electron spectrometer. The results are shown in Supplementary Figure 1. Indeed, we observe similar structures around 48 and 66 eV as highlighted by the red shaded areas (42-55 eV and 60-72 eV) matching well with the previous reported ETMD peaks for Al^{3+} and confirming the validity of our method.

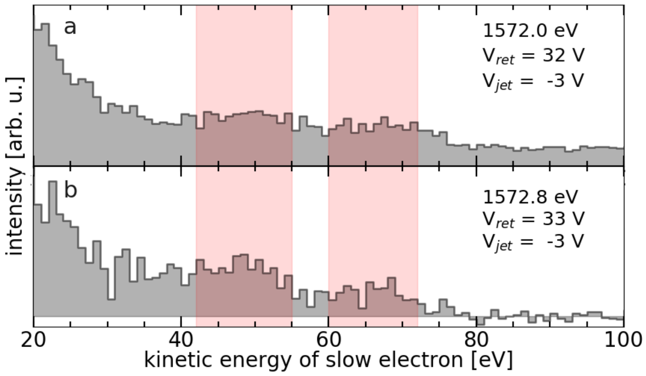

Supplementary Figure 1: Difference spectra after Al^{3+} 1s ionization. Difference spectra from double-electron coincidences of a 2 M $AlCl_3$ solution at two slightly different exciting-photon energies and retardation bias voltages. The coincidence condition was set for the unresolved Auger electron emitted after Al^{3+} 1s ionization. The variant (1) of background subtraction was used (see text) and the curves were previously normalized to their maximum. The red shaded regions indicate where ETMD structures have been observed recently[6]. These spectra were recorded at PETRA III. Source data are provided as a Source Data file.

Supplementary Note 3

We recorded several double- and triple-electron coincidence spectra for 2p, 2s, and 1s ionization of solvated Mg^{2+} ions at different exciting-photon energies. The difference spectra after 2p ionization resulting from double coincidences are illustrated in Supplementary Figure 2. The spectra are measured at an exciting-photon energy of 145 eV (a and d) and 175 eV (b and c). The green curve [Supplementary Figure 2(a)] is identical to the spectrum in Fig. 3(b). Two features (7-16 eV and 20-30 eV) highlighted in the red shaded regions can be identified, which do not shift with varying exciting-photon energy. Those features are attributed to ETMD electrons (see main article).
Supplementary Figures 2(a) and (b) were recorded with equal retardation and jet bias resulting in the low-energy cutoff around 5 eV. Supplementary Figure 2(c) was measured with no retardation voltage resulting in a low-energy cutoff close to 0 eV and a strong increase of the signal towards lower kinetic energies, somewhat masking the two features. Note that an accurate calibration from time-of-flight values to kinetic energies is challenging for very slow electrons, since those are hardly visible in the background of slow electrons from the liquid [1–3].

Supplementary Figure 2: Difference spectra after Mg^{2+} 2p ionization. Difference spectra from double (a to c) and triple-electron coincidences (d) of a 3 M $MgCl_2$ solution at different exciting-photon energies and retardation bias voltages. a and d were recorded at an exciting-photon energy of 145 eV, b and c at 175 eV. The coincidence condition was set for all spectra for the first electron being the Mg^{2+} 2p photoelectron. The method (1) of background subtraction was used after normalization to the curve maximum. The red-shaded regions highlight two features present in the spectra from a to c. The hatched curve in d corresponds to the spectrum of the third and the filled curve to the spectrum of the second electron of the triple coincidences. All spectra were recorded at BESSY II. The green spectrum a is identical to the spectrum in Fig. 3(b). Source data are provided as a Source Data file.

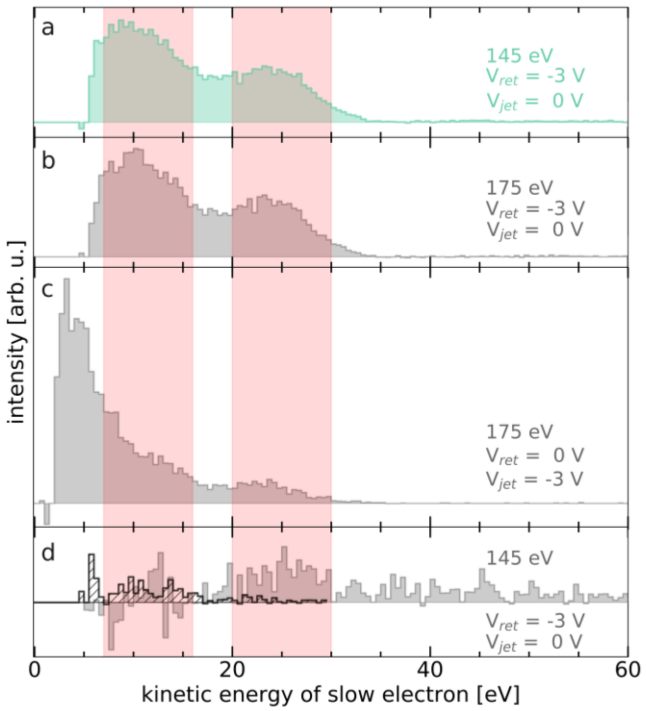

Supplementary Figures 3 and 4 present the difference spectra after Mg^{2+} 1s ionization recorded at BESSY II and PETRA III, respectively. The three spectra in Supplementary Figure 3 were measured at three different photon energies and obtained from analysis of double-electron coincidences. The magenta spectrum [Supplementary Figure 3(b)] is identical to the curve in Fig. 3(c). Supplementary Figure 3(a) was obtained by applying the coincidence condition to the Auger electron emitted after the Mg^{2+} 1s ionization, that is why the 1s photoelectron appears at 57 eV kinetic energy. For b and c, the coincidence condition was set to the Mg^{2+} 1s photoelectron. Note that the negative intensities are artefacts from the background normalization and subtraction procedure with no physical meaning. Highlighted in red-shaded regions (16-29 eV and 34-46 eV) are two features observed independently of the photon energy or coincidence condition and associated with various ICD and ETMD processes (see main article).

Supplementary Figure 3: Difference spectra after Mg^{2+} 1s ionization, recorded at BESSY II. Difference spectra from double-electron coincidence datasets of a $MgCl_2$ solution at different exciting-photon energies and applied bias voltages. The data in panel a was measured with a 2 M $MgCl_2$ solution and the data in panels b and c with a 3 M $MgCl_2$ solution. a, b, and c were recorded at 1367, 1387, and 1397 eV, respectively. The difference spectrum in panel a was obtained with the coincidence condition set to the Auger electron emitted after the 1s ionization and variant (1) of background subtraction after normalization to the curve maximum. The spectra in b and c result from the coincidence condition set to the Mg^{2+} 1s photoelectron and variant (2) of background subtraction after normalization to the curve maximum. The red-shaded regions highlight two features present in all three spectra. These spectra were recorded at BESSY II. The magenta spectrum b is identical to the spectrum in Fig 3(c). Source data are provided as a Source Data file.

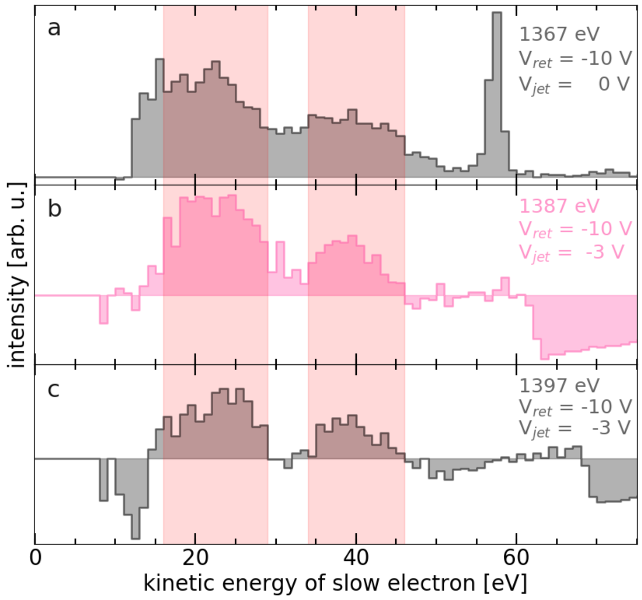

The difference spectra shown in Supplementary Figure 4 originate from double- and triple-electron coincidences with distinct photon energies near the Mg^{2+} 1s threshold (around 1309.9 eV[7]). The red shaded regions are identical to Supplementary Figure 3. All three datasets are structured, but the features of interest seem to be notably less pronounced in the double-coincidence spectra in Supplementary Figure 4 (measured at PETRA III) compared to the double-coincidence spectra from Fig. 3 (measured at BESSY II).

The main difference of the spectra lies in the exciting-photon energy. The spectra from PETRA III (Supplementary Figure 4) were obtained very close to the Mg^{2+} 1s edge, whereas the spectra from BESSY II (Supplementary Figure 3) were recorded well above the threshold. Consequently, coincidences with photoelectrons are observed at very low kinetic energies for the PETRA III measurements [indicated by blue solid (a) and green solid lines (b and c) in Supplementary Figure 4]. This somewhat reduces the significance of all other features, which is not a problem at an excess energy around 57 eV [Supplementary Figure 3(a)]. The triple-electron coincidence spectrum needs to be interpreted with caution. No conclusion can be drawn about the photoelectron – ICD/ETMD electron ratio. This is due to the fact that the photoelectron is partly recorded in the second or third electron spectra (e.g., if one electron was missed) and both spectra are subtracted and scaled independently from each other. Summarizing, it is strongly convincing to obtain the same structures assigned to ICD/ETMD independently of the exciting-photon energy, synchrotron facility, and coincidence condition.

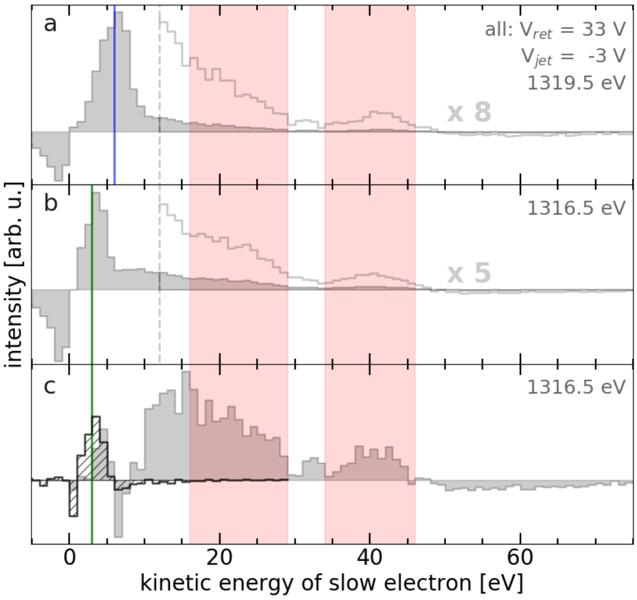

Supplementary Figure 4: Difference spectra after Mg^{2+} 1s ionization recorded at PETRA III. Difference spectra from double- (a and b) and triple-electron (c) coincidences of a 4 M $MgCl_2$ solution at different photon energies and equal retardation bias voltages. The filled curve in panel c corresponds to the spectrum of the second and the hatched curve to the spectrum of the third electron of the triple coincidences. The coincidence conditions were set for all spectra for the Auger electron emitted after Mg^{2+} 1s ionization and variant (1) of background subtraction after normalization to the curve maximum was used. Red-shaded are two regions highlighting the kinetic energies of main interest, identical to Supplementary Figure 3. The blue solid line in a and green solid lines in b and c (around 3 and 6 eV) indicate the shift of the Mg^{2+} 1s photoelectron peak. Above the gray dashed line at 12 eV in a and b the double-electron spectra are multiplied by 8 and 5, respectively, and shown as light gray curves. The spectra were recorded at PETRA III. Source data are provided as a Source Data file.

In Supplementary Figure 5, we present difference spectra for the ionization of the Mg^{2+} 2s orbital at two different exciting-photon energies obtained from double- and triple-electron coincidences. Supplementary Figures 5(a) and (c) were recorded at a photon energy of 145 eV and (b) and (d) at 175 eV. The blue spectrum (a) is identical to Fig. 4(a). The red-shaded region highlights a feature assigned to ICD (see main article), present in all spectra and not shifting with exciting-photon energy. For ionization of the 2s orbital in total three electrons are expected to be emitted: the 2s photoelectron, the ICD electron, and one ETMD electron (see Fig. 2). Therefore, a maximum around 20 eV should appear also in the triple-electron coincidence dataset which can indeed be found. In contrast, for the 2p ionization only two electrons should be emitted (one 2p photoelectron and one ETMD electron). Here, the triple-electron coincidences do not show any significant structure compared to the background signal [Supplementary Figure 2(d)].

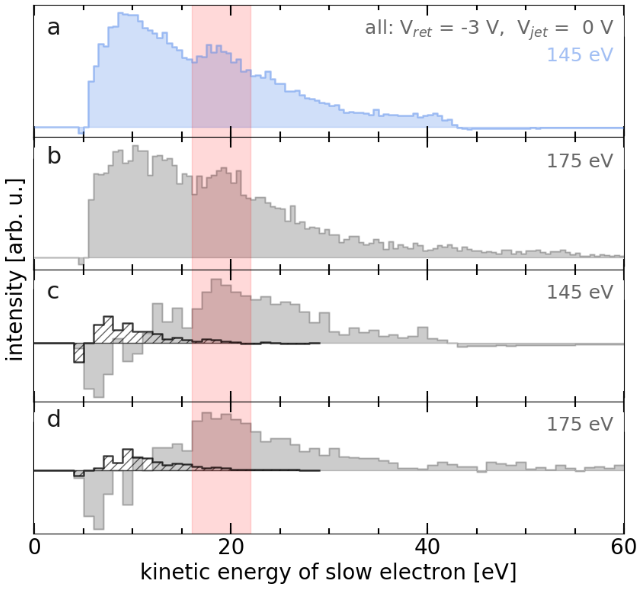

Supplementary Figure 5: Difference spectra after Mg^{2+} 2s ionization. Difference spectra from double- and triple-electron coincidences of a 3 M $MgCl_2$ solution at different photon energies and equal retardation bias voltages. Panels a and c were recorded at an exciting-

photon energy of 145 eV, b and d at 175 eV. The upper two spectra (a and b) correspond to double- and the lower spectra (c and d) to triple-electron coincidences. In the latter case, the filled curves show the spectra of the second and the hatched curves the spectra of the third electron of the triple coincidences. The coincidence conditions were set for all spectra to the Mg^{2+} 2s photoelectron and variant (1) of background subtraction after normalization to the curve maximum was applied. The red-shaded region highlights a feature present in all four datasets. The spectra were recorded at BESSY II. The blue spectrum a is identical to the blue spectrum from Fig. 4(a). Source data are provided as a Source Data file.

Supplementary References

1. Malerz, S. *et al.* Low-energy constraints on photoelectron spectra measured from liquid water and aqueous solutions. *Phys. Chem. Chem. Phys.* **23**, 8246–8260 (2021).
2. Gadeyne, T., Zhang, P., Schild, A. & Wörner, H. J. Low-energy electron distributions from the photoionization of liquid water: a sensitive test of electron mean free paths. *Chem. Sci.* **13**, 1675–1692 (2022).
3. Signorell, R. & Winter, B. Photoionization of the aqueous phase: clusters, droplets and liquid jets. *Phys. Chem. Chem. Phys.* **24**, 13438–13460 (2022).
4. Unger, I. *et al.* Observation of electron-transfer-mediated decay in aqueous solution. *Nat. Chem.* **9**, 708–714 (2017).
5. Zhang, P., Perry, C., Luu, T. T., Matselyukh, D. & Wörner, H. J. Intermolecular Coulombic Decay in Liquid Water. *Phys. Rev. Lett.* **128**, 133001 (2022).
6. Gopakumar, G. *et al.* Radiation damage by extensive local water ionization from two-step electron-transfer-mediated decay of solvated ions. *Nat. Chem.* **15**, 1408–1414 (2023).
7. Gopakumar, G. *et al.* Probing aqueous ions with non-local Auger relaxation. *Phys. Chem. Chem. Phys.* **24**, 8661–8671 (2022).
